# DCTN2 and PAX8 levels in prostatic carcinoma tissues and their relationship with clinicopathological features and prognosis

**DOI:** 10.3389/fonc.2026.1800547

**Published:** 2026-06-29

**Authors:** Xiaolong He, Haiqin Zhu, Difu Fan, Xiaolin Deng, Yongming Huang

**Affiliations:** 1 Department of Urology, The Affiliated Ganzhou Hospital, Jiangxi Medical College, Nanchang University, Ganzhou, China; 2 Department of Personnel, The Affiliated Ganzhou Hospital, Jiangxi Medical College, Nanchang University, Ganzhou, China

**Keywords:** clinicopathological features, dynactin 2, paired box gene 8, prognosis, prostatic carcinoma

## Abstract

**Objective:**

To investigate the levels of dynactin 2 (DCTN2) and paired box gene 8 (PAX8) in prostatic carcinoma (PCa) tissues and their relationship with clinicopathological features and prognosis.

**Methods:**

A total of 103 patients with PCa admitted between February 2020 and February 2022 were selected as the study subjects. Immunohistochemical staining was used to detect the expression levels of DCTN2 and PAX8 in tissue samples. The associations of DCTN2 and PAX8 expression in cancer tissues with clinicopathological characteristics and prognosis were analyzed.

**Results:**

The positive expression rates of DCTN2 and PAX8 were higher in PCa tissues than in adjacent tissues (P<0.05). DCTN2 and PAX8 expression in cancer tissues was associated with clinicopathological features (P<0.05). DCTN2 and PAX8 expression in cancer tissues was related to tumor differentiation grade, lymph node metastasis, clinical stage, distant metastasis, preoperative PSA, and Gleason score (P<0.05). Tumor differentiation, lymph node metastasis, clinical stage, distant metastasis, preoperative PSA, Gleason score, DCTN2, and PAX8 differed significantly among patients with different prognoses (P<0.05). The 3-year progression-free survival rate of positive DCTN2 and PAX8 expression was lower than that of negative DCTN2 and PAX8 expression (log-rank χ²=9.656, 9.288, P = 0.002, 0.002). Lymph node metastasis, clinical stage, Gleason score, DCTN2 and PAX8 expression in tissues were factors affecting poor prognosis in PCa patients (P<0.05).

**Conclusion:**

The positive expression rates of DCTN2 and PAX8 were elevated in PCa tissues, and both markers were associated with the clinicopathological features and prognosis of patients.

## 1 Introduction

Prostate cancer (PCa) is a common malignant tumor in men, with clinical symptoms such as frequent urination, urgency, dysuria, and difficulty urinating. In recent years, the incidence and mortality of PCa have gradually increased (1, 2). The exact causes of PCa are not fully understood but may be related to age, heredity, living environment, obesity, and hormone levels in the body. Currently, the preferred treatment for PCa is radical surgery followed by endocrine therapy. However, some patients experience postoperative recurrence or tumor progression, which seriously affects their quality of life and health (3, 4). A comprehensive investigation of biomarkers associated with the occurrence and progression of PCa is important for elucidating its pathogenesis and identifying potential targets for therapy.

Dynactin 2 (DCTN2) is a cancer-related protein that is abnormally expressed in various types of cancer tissues, such as liver cancer. Overexpression of DCTN2 is associated with reduced survival rates in patients (5). Paired box gene 8 (PAX8) is a transcription factor that may be involved in the occurrence of thyroid cancer by regulating the proliferation and differentiation of thyroid cells. PAX8 can interact with other genes to jointly regulate the development of thyroid cells (6). Based on previous literature and studies on the molecular mechanisms of PCa, this study hypothesized *a priori* that DCTN2 and PAX8 are highly expressed in PCa tissues, and that their abnormally high expression is closely associated with high-risk clinicopathological features, including a high Gleason score, lymph node metastasis, distant metastasis, and high preoperative PSA levels. Moreover, both DCTN2 and PAX8 may be independent risk factors for poor prognosis in patients with PCa.

## 2 Subjects and methods

### 2.1 Subjects

**Figure 1:**
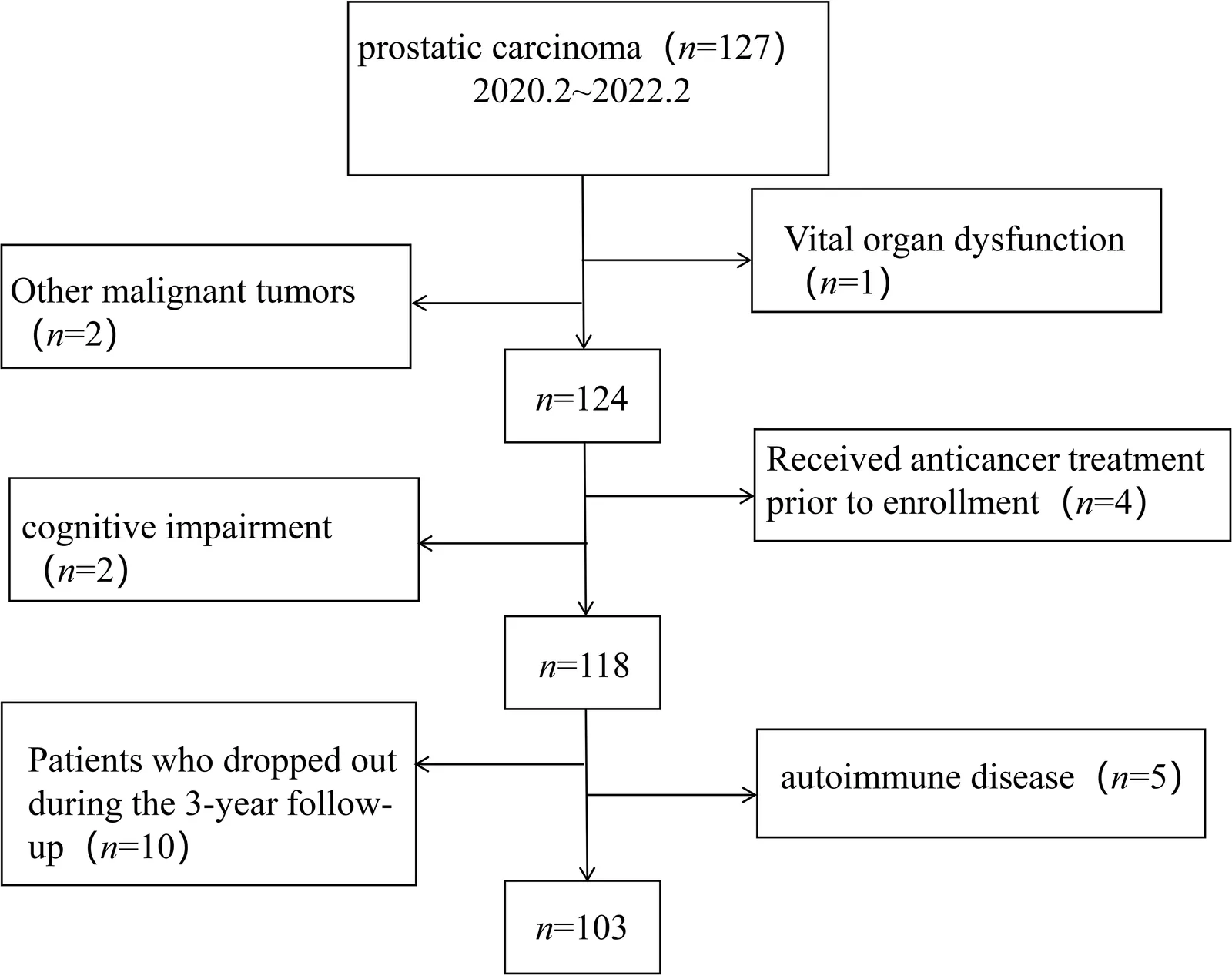
Flowchart of case collection.

The sample size was calculated using the formula N = Z² × [P × (1 − P)]/E². When Z = 1.64, E = 10%, and P = 0.5, the calculated sample size was 67. Considering a 10% loss to follow-up rate, at least 74 patients were required. In the present study, 103 patients were included, indicating that the study had a certain degree of statistical power. A total of 103 PCa patients admitted between February 2020 and February 2022 were selected as the study subjects. Inclusion criteria: ① met the diagnostic criteria for PCa (7); ② all underwent radical surgical treatment. Exclusion criteria: ① dysfunction of important organs; ② other malignant tumors; ③ received anticancer treatment before enrollment; ④ cognitive dysfunction; ⑤ autoimmune diseases. See **Figure 1**.

### 2.2 Methods

#### 2.2.1 Data collection

Data were obtained from the hospital medical record system, including age, tumor diameter, tumor differentiation, lymph node metastasis, clinical stage, distant metastasis, smoking history, alcohol consumption history, preoperative prostate-specific antigen (PSA), and Prostate Cancer Gleason Scoring System (Gleason score).

#### 2.2.2 Immunohistochemical staining to detect DCTN2 and PAX8 levels in tissues

Cancer tissues and adjacent tissues (>1 cm away from the edge of the cancer tissue and confirmed as normal prostate tissue by histopathological examination) were collected from PCa patients during surgery. The tissue samples used for immunohistochemical analysis were fixed with formaldehyde, embedded in paraffin, and sectioned. Immunohistochemical staining was then performed to detect the expression levels of DCTN2 and PAX8 in cancer tissues and adjacent tissues.

Criteria for result determination: Five clear fields of view were randomly selected (under high magnification). (1) Scoring was based on the percentage of positive cells in the field of view: ≤10%, 11%-49%, 50%-74%, and ≥75% corresponded to 0–3 points, respectively; (2) colorless, light yellow, brownish yellow, and brown corresponded to 0–3 points, respectively; the final score was calculated as (1) × (2). Scores <4 were considered negative, and scores ≥4 were considered positive.

#### 2.2.3 Follow-up and prognosis assessment

Patients were followed up for 3 years until February 1, 2025, via telephone follow-up and outpatient re-examinations. Follow-up was conducted every 3 months in the first postoperative year and every 6 months in the second to third postoperative years. Tumor progression was recorded. Tumor progression included local recurrence/metastasis, biochemical recurrence, or death. Progression-free survival was defined as the interval from patient discharge to tumor progression. Follow-up ended at the end of the follow-up period or when tumor progression occurred.

### 2.3 Statistical analysis

SPSS 26.0 was used for data processing. Count data were expressed as n (%), and the chi-square test was performed. Measurement data conforming to a normal distribution were expressed as the mean ± standard deviation $\bar{x}\pm s$, and the t-test was performed. The Kaplan-Meier method was used to analyze the relationship between DCTN2 and PAX8 expression and patient prognosis. Multivariate Cox regression analysis was used to analyze influencing factors. P < 0.05 indicated a statistically significant difference.

## 3 Results

### 3.1 Comparison of immunohistochemical results of DCTN2 and PAX8 in cancer tissue and adjacent tissue

**Figure 2:**
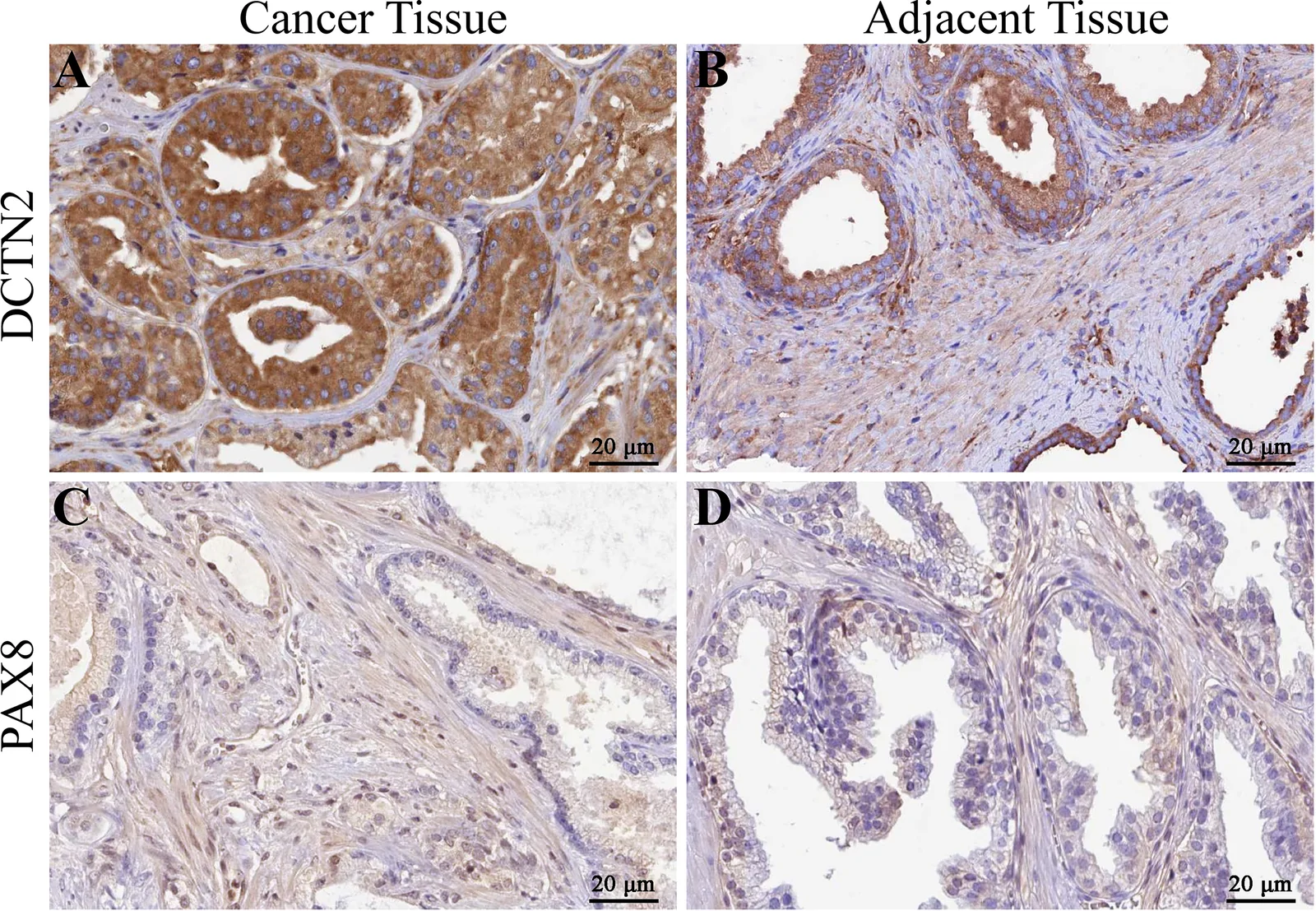
Immunohistochemistry for DCTN2 and PAX8 in cancer tissue and adjacent tissue. **(A)** DCTN2 in cancer tissue; **(B)** DCTN2 in adjacent tissue; **(C)** PAX8 in cancer tissue; **(D)** PAX8 in adjacent tissue.

Immunohistochemical staining showed that positive expression of both DCTN2 and PAX8 was brownish and mainly localized in the cytoplasm. The positive expression rates of DCTN2 and PAX8 in PCa tissues were significantly higher than those in adjacent tissues (P<0.05), as shown in **Table 1**, **Figure 2**.

**Table 1:** Comparison of Immunohistochemical Results of DCTN2 and PAX8 in Different Tissues [n(%)].

Groups	Number of examples	DCTN2		PAX8	
		Positive	Negatives	Positive	Negatives
cancer tissue	103	62(60.19)	41(39.81)	66(64.08)	37(35.92)
paraneoplastic tissue	103	29(28.16)	74(71.84)	32(31.07)	71(68.93)
χ2		21.437		22.500	
P		<0.001		<0.001	

### 3.2 Correlation between DCTN2 and PAX8 expression and clinicopathological features in cancer tissues

There were no significant differences in DCTN2 and PAX8 expression among patients with different age groups, tumor diameters, smoking history, and alcohol consumption history (P>0.05). However, there were statistically significant differences in DCTN2 and PAX8 expression among patients with different tumor differentiation grades, lymph node metastasis, clinical stages, distant metastasis, preoperative PSA levels, and Gleason scores (P<0.05), as shown in **Table 2**.

**Table 2:** Correlation between DCTN2 and PAX8 expression and clinicopathological features in cancer tissues [n(%)].

Clinicopathological feature	Number of cases	DCTN2		χ²	P	PAX8		χ²	P
		Positive(n = 62)	Negative(n = 41)			Positive(n = 66)	Negative(n = 37)		
age				2.471	0.116			2.650	0.104
>60 year	53	28(52.83)	25(47.17)			30(56.60)	23(43.40)		
≤60 year	50	34(68.00)	16(32.00)			36(72.00)	14(28.00)		
Tumor diameter				0.674	0.412			2.400	0.121
<2 cm	35	23(65.71)	12(34.29)			26(74.29)	9(25.71)		
≥2 cm	68	39(57.35)	29(42.65)			40(58.82)	28(41.18)		
tumor differentiation				10.116	0.006			10.387	0.006
high differentiation	51	23(45.10)	28(54.90)			25(49.02)	26(50.98)		
moderate differentiation	23	16(69.57)	7(30.43)			17(73.91)	6(26.09)		
poor differentiation	29	23(79.31)	6(20.69)			24(82.76)	5(17.24)		
lymph node metastasis				14.099	<0.001			14.130	<0.001
yes	56	43(76.79)	13(23.21)			45(80.36)	11(19.64)		
no	47	19(40.43)	28(59.57)			21(44.68)	26(55.32)		
clinical stage				4.573	0.032			5.784	0.016
Stage I~II	65	34(52.31)	31(47.69)			36(55.38)	29(44.62)		
Stage III	38	28(73.68)	10(26.32)			30(78.95)	8(21.05)		
distant metastasis				4.744	0.029			7.986	0.005
yes	21	17(80.95)	4(19.05)			19(90.48)	2(9.52)		
no	82	45(54.88)	37(45.12)			47(57.32)	35(42.68)		
smoking history				0.130	0.718			2.190	0.139
yes	60	37(61.67)	23(38.33)			42(70.00)	18(30.00)		
no	43	25(58.14)	18(41.86)			24(55.81)	19(44.19)		
drinking history				0.602	0.438			0.233	0.630
yes	58	33(56.90)	25(43.10)			36(62.07)	22(37.93)		
no	45	29(64.44)	16(35.56)			30(66.67)	15(33.33)		
Preoperative PSA				7.870	0.005			11.430	0.001
>15 ng/mL	45	34(75.56)	11(24.44)			37(82.22)	8(17.78)		
5~15 ng/mL	58	28(48.28)	30(51.72)			29(50.00)	29(50.00)		
Gleason score				5.964	0.015			6.498	0.011
≥7 points	74	50(67.57)	24(32.43)			53(71.62)	21(28.38)		
<7 points	29	12(41.38)	17(58.62)			13(44.83)	16(55.17)		

### 3.3 Differences in clinical data and DCTN2 and PAX8 expression among patients with different prognoses

There were significant differences in tumor differentiation, lymph node metastasis, clinical stage, distant metastasis, preoperative PSA, Gleason score, and DCTN2 and PAX8 expression among patients with different prognoses (P<0.05), as shown in **Table 3**.

**Table 3:** Differences in clinical data and DCTN2 and PAX8 expression among patients with different prognoses [n(%)].

Variable	Poor prognosis group(n = 29)	Good prognosis group(n = 74)	*t/*χ²	P
tumor differentiation			39.088	<0.001
poor differentiation	21(72.41)	8(10.81)		
Moderate and high differentiation	8(27.59)	66(89.19)		
lymph node metastasis			7.516	0.006
yes	22(75.86)	34(45.95)		
no	7(24.14)	40(54.05)		
clinical stage			17.834	<0.001
Stage I~II	9(31.03)	56(75.68)		
Stage III	20(68.97)	18(24.32)		
distant metastasis			30.089	<0.001
yes	16(55.17)	5(6.76)		
no	13(44.83)	69(93.24)		
Preoperative PSA			16.983	<0.001
>15 ng/mL	22(75.86)	23(31.08)		
5~15 ng/mL	7(24.14)	51(68.92)		
Gleason score			4.116	0.042
≥7 points	25(86.21)	49(66.22)		
<7 points	4(13.79)	25(33.78)		
DCTN2			8.577	0.003
positive	24(82.76)	38(51.35)		
negative	5(17.24)	36(48.65)		
PAX8			8.587	0.003
positive	25(86.21)	41(55.41)		
negative	4(13.79)	33(44.59)		

### 3.4 Correlation between DCTN2 and PAX8 expression and prognosis

**Figure 3:**
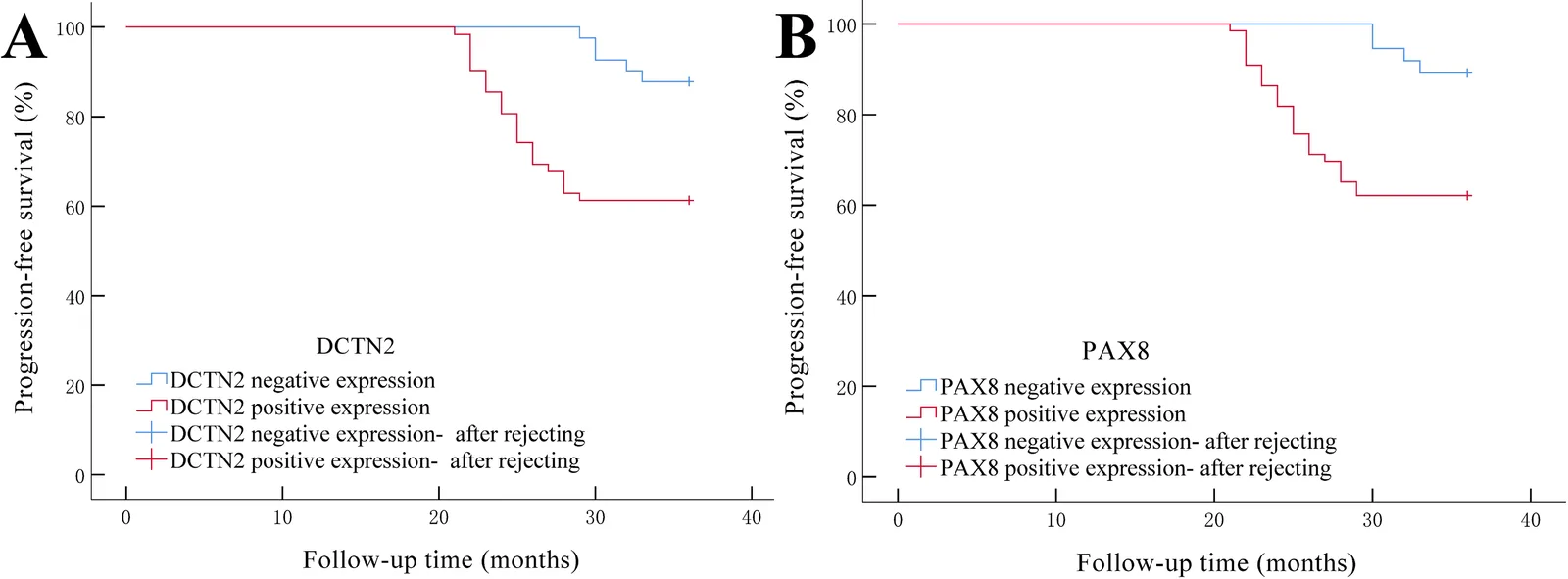
Kaplan-Meier analysis of the relationship between DCTN2 and PAX8 expression and prognosis. **(A)** Relationship between DCTN2 expression and prognosis; **(B)** Relationship between PAX8 expression and prognosis.

Kaplan-Meier analysis of the relationship between DCTN2 and PAX8 expression and patient prognosis showed tumor progression in 29 cases, with a 3-year progression-free survival rate of 71.84% (74/103). The 3-year progression-free survival rate for positive DCTN2 expression was 61.29% (38/62), which was lower than the 87.80% (36/41) for negative DCTN2 expression, and the difference was statistically significant (log-rank χ²=9.656, P = 0.002). The 3-year progression-free survival rate for positive PAX8 expression was 62.12% (41/66), which was lower than the 89.19% (33/37) for negative PAX8 expression, and the difference was statistically significant (log-rank χ²=9.288, P = 0.002). See **Figure 3**.

### 3.5 Cox regression analysis of poor prognostic factors in PCa patients

Cox regression analysis was performed with the prognosis of patients with PCa as the dependent variable (tumor progression=1, no tumor progression=0) and tumor differentiation, lymph node metastasis, clinical stage, distant metastasis, preoperative PSA, Gleason score, and DCTN2 and PAX8 expression in tissues as independent variables (assignments shown in **Table 4**). The results showed that lymph node metastasis, clinical stage, Gleason score, and DCTN2 and PAX8 expression in tissues were independent risk factors for poor prognosis in PCa patients (P<0.05), as shown in **Table 5**.

**Table 4:** Assignment explanation.

Variable	Assignment
tumor differentiation	poor differentiation=1, Moderate and high differentiation=0
lymph node metastasis	yes=1, no=0
clinical stage	Stage III = 1, Stage I~II=0
distant metastasis	yes=1, no=0
Preoperative PSA	>15 ng/mL=1, 5~15 ng/mL=0
Gleason score	≥7 points=1, <7 points=0
DCTN2	positive=1, negative=0
PAX8	positive=1, negative=0

**Table 5:** Factors influencing poor prognosis in PCa patients analyzed by multivariate Cox analysis.

Variable	β	S.E.	HR	Wald χ^χ²^	P	95% CI
tumor differentiation	0.823	0.503	2.278	2.679	0.102	0.850-6.105
lymph node metastasis	1.584	0.487	4.875	10.581	0.001	1.877-12.662
clinical stage	1.543	0.378	4.677	16.655	<0.001	2.229-9.811
distant metastasis	0.937	0.511	2.553	3.364	0.067	0.938-6.951
Preoperative PSA	0.759	0.462	2.136	2.699	0.100	0.864-5.283
Gleason score	1.477	0.287	4.381	26.495	<0.001	2.496-7.689
DCTN2	1.676	0.267	5.342	39.384	<0.001	3.165-9.015
PAX8	1.854	0.229	6.385	65.543	<0.001	4.076-10.002

## 4 Discussion

PCa is a disease with diverse clinical and molecular characteristics and is the most common type of cancer in men. Currently, the incidence and mortality rates of PCa are increasing globally, and there are many influencing factors, including genetic factors, family history, racial background, obesity, alcohol consumption, and smoking (8, 9). Early PCa progresses relatively slowly, and most individuals do not have typical symptoms. However, when cancer cells proliferate or spread rapidly, patients may experience symptoms such as difficulty urinating (10, 11). Currently, the most common clinical treatment for PCa is radical surgery, but some patients experience recurrence, affecting their daily lives (12, 13). Therefore, exploring biomarkers related to the occurrence and development of PCa is helpful for clinically revealing the pathogenesis of PCa and identifying new directions for targeted therapy. This study detected the expression levels of DCTN2 and PAX8 in tissues of PCa patients and analyzed their relationship with the patients’ clinicopathological features and prognosis, with the aim of assisting clinical evaluation of patient prognosis.

DCTN2 is a multisubunit complex that plays an important role in various cellular functions and can participate in cytoskeleton remodeling and mitosis by activating dynein (14, 15). The expression of DCTN2 in hepatocellular carcinoma tissues is significantly higher than that in non-tumor tissues, and silencing DCTN2 can significantly inhibit the proliferation and metastasis of tumor cells. The mechanism by which DCTN2 participates in hepatocellular carcinoma is related to the AKT pathway (5). The results of this study showed that the positive expression rate of DCTN2 in PCa tissues was higher than in adjacent tissues, suggesting that DCTN2 expression is closely related to the occurrence of PCa, and DCTN2 may also participate in the occurrence of PCa through the AKT pathway. The specific mechanism requires verification in basic experimental studies.

PAX8 belongs to the paired-box gene family and is a transcription factor related to the development of various tissues and organs and is especially important in the development of the thyroid gland. In thyroid follicular tumors, PAX8 exhibits positive nuclear staining and can be used to distinguish thyroid tumors from other types of tumors (16, 17). Studies have shown that the transcription factor PAX8 promotes angiogenesis in ovarian cancer by interacting with the developmental transcription factor SOX17 (18). Previous studies have shown that PAX8 exhibits positive expression in 40 types of cancer, with positive rates, from high to low, in follicular thyroid tumors, gynecological cancers, kidney tumors, and urothelial tumors. In non-invasive urothelial carcinoma, high PAX8 expression is associated with low tumor grade (19). PAX8 can affect the glycolysis and proliferation of pancreatic cancer cells by regulating the Notch1 signaling pathway (20). This study found that the positive expression rate of PAX8 in PCa tissues was higher than that in adjacent tissues, indicating that PAX8 may be related to the occurrence of PCa, and PAX8 may also participate in the occurrence and development of PCa by regulating specific signaling pathways.

Exploring the clinicopathological features of cancer is helpful for formulating clinical treatment plans, understanding the pathogenesis of cancer, and evaluating patient prognosis. This study found that DCTN2 and PAX8 expression were closely related to tumor differentiation grade, lymph node metastasis, clinical stage, distant metastasis, preoperative PSA, and Gleason score, suggesting that DCTN2 and PAX8 may be related to the progression of PCa. Preoperative PSA is an important indicator for PCa screening and diagnosis, and the Gleason score is an important indicator for evaluating the malignancy of PCa. Both are closely related to patient prognosis. The significant difference in DCTN2 and PAX8 expression in different preoperative PSA levels and Gleason scores indicates that DCTN2 and PAX8 expression may be closely related to the prognosis of PCa patients, and more patients will be included for further analysis and verification in the future.

Prognostic analysis showed that the 3-year progression-free survival rates of PCa patients with positive DCTN2 and PAX8 expression were significantly lower than those with negative expression, suggesting that DCTN2 and PAX8 expression are closely related to patient prognosis. There were significant differences in tumor differentiation, lymph node metastasis, clinical stage, distant metastasis, preoperative PSA, Gleason score, and DCTN2 and PAX8 expression among patients with different prognoses. Further analysis of prognostic factors showed that, in addition to lymph node metastasis, clinical stage, and Gleason score, DCTN2 and PAX8 expression levels were also influencing factors for poor prognosis in PCa patients. This further confirms the importance of DCTN2 and PAX8 in PCa prognosis. Clinicians should closely monitor PCa patients with positive DCTN2 and PAX8 expression, as these patients may have a higher risk of poor prognosis. The specific mechanisms by which DCTN2 and PAX8 participate in the occurrence and development of PCa need further exploration.

In summary, the positive expression rates and expression levels of DCTN2 and PAX8 in PCa tissues are significantly increased, and both are closely related to the clinicopathological features and prognosis of patients. The limitations of this study are the small sample size and the limited generalizability of the research results.

## Data Availability

The original contributions presented in the study are included in the article/supplementary material. Further inquiries can be directed to the corresponding author.
